# Hype vs Reality in the Integration of Artificial Intelligence in Clinical Workflows

**DOI:** 10.2196/70921

**Published:** 2025-12-12

**Authors:** Alaa Abd-Alrazaq, Barry Solaiman, Yosra Magdi Mekki, Dena Al-Thani, Faisal Farooq, Metab Alkubeyyer, Mohamed Ziyad Abubacker, Rawan AlSaad, Sarah Aziz, Ahmed Serag, Rajat Thomas, Javaid Sheikh, Arfan Ahmed

**Affiliations:** 1 AI Center for Precision Health, Weill Cornell Medical College in Qatar, Education City, Doha, P.O. Box 24144, Qatar, 97444928826; 2 College of Law, Hamad bin Khalifa University, Doha, Qatar; 3 Department of Basic Medical Sciences, College of Medicine, QU Health, Qatar University, Doha, Qatar; 4 College of Science and Engineering, Hamad bin Khalifa University, Doha, Qatar; 5 Qatar Computational Research Institute (QCRI), Hamad Bin Khalifa University, Doha, Qatar; 6 Department of Radiology and Medical Imaging, King Khalid University Hospital, King Saud University, Riyadh, Saudi Arabia; 7 Department of Radiology, Al Ahli Hospital, Doha, Qatar; 8 AI Innovation Lab, Weill Cornell Medical College, Doha, Qatar; 9 Weill Cornell Medical College, Doha, Qatar

**Keywords:** artificial intelligence, AI, clinical workflow, challenges, solutions, technology, human factors, ethics, regulation, health care

## Abstract

Artificial intelligence (AI) has the capacity to transform health care by improving clinical decision-making, optimizing workflows, and enhancing patient outcomes. However, this potential remains limited by a complex set of technological, human, and ethical barriers that constrain its safe and equitable implementation. This paper argues for a holistic, systems-based approach to AI integration that addresses these challenges as interconnected rather than isolated. It identifies key technological barriers, including limited explainability, algorithmic bias, integration and interoperability issues, lack of generalizability, and difficulties in validation. Human factors such as resistance to change, insufficient stakeholder engagement, and education and resource constraints further impede adoption, whereas ethical and legal challenges related to liability, privacy, informed consent, and inequity compound these obstacles. Addressing these issues requires transparent model design, diverse datasets, participatory development, and adaptive governance. Recommendations emerging from this synthesis are as follows: (1) establish standardized international regulatory and governance frameworks; (2) promote multidisciplinary co-design involving clinicians, developers, and patients; (3) invest in clinician education, AI literacy, and continuous training; (4) ensure equitable resource allocation through dedicated funding and public-private partnerships; (5) prioritize multimodal, explainable, and ethically aligned AI development; and (6) focus on long-term evaluation of AI in real-world settings to ensure adaptive, transparent, and inclusive deployment. Adopting these measures can align innovation with accountability, enabling health care systems to harness AI’s transformative potential responsibly and sustainably to advance patient care and health equity.

## Introduction

Artificial intelligence (AI) is reshaping clinical health care, yet real-world uptake remains limited. The gap between hype and reality stems from fragmented integration that treats technology, people, and ethics as separate tracks. This viewpoint argues that responsible adoption requires a holistic integration framework that advances technological robustness, human alignment, and ethical and regulatory accountability together. Progress in any 1 domain without the others undermines impact; therefore, coordinated advancement across all 3 is needed to translate promise into clinical value. AI tools already influence high-stakes clinical decisions in imaging, triage, treatment selection, and remote monitoring. Their rapid diffusion, sometimes ahead of regulation and robust evidence, underscores the need for a principled, holistic integration framework. Prior reviews catalog challenges but rarely explain how the domains interact or how to coordinate solutions across stakeholders [1-4]. This paper contributes by leading the conversation from what the challenges are to how they can be collectively resolved.

**Figure 1.**
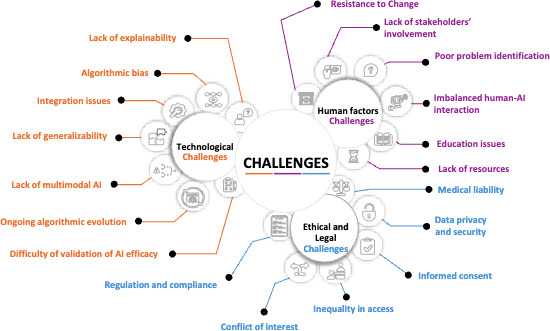
Challenges of integrating artificial intelligence (AI) in clinical workflows.

Figure 1 summarizes three interlocking domains of AI integration: technological, human, and ethical and legal. Each domain includes principal challenges, for example, explainability and bias; interoperability and generalizability; workforce engagement and training; and liability, privacy, consent, equity, and conflicts of interest. Together, these dependencies determine whether AI becomes a trusted clinical partner or remains a research accessory. To preserve concision, detailed technical explanations, case examples, and jurisdictional analyses are provided in Appendix A in Multimedia Appendix 1. The translation gap reflects misalignment between rapid technical progress and slower social, ethical, and institutional adaptation. Four principles guide the analysis: validation parity (AI systems should meet evidence standards comparable to drugs and devices), human and AI complementarity (tools should augment rather than replace clinician judgment), transparency and accountability (models and processes must be traceable, with clear responsibility across developers, institutions, and regulators), and equitable infrastructure (fair access to data, talent, and computing so that adoption does not widen disparities). These principles frame each domain discussed next and anchor the targeted solutions.

We write for clinicians, policymakers, ethicists, and technologists and move beyond describing obstacles to explain why partial fixes fail and how coordinated approaches can succeed. The paper aims to clarify how technical design links to human trust and ethical legitimacy; to translate this into strategies that turn prototypes into regulated clinical tools; and to offer concise, actionable recommendations for policy, practice, and research. We address 3 domains aligned with Figure 1 (ie, technological foundations, human and organizational dimensions, and ethical and regulatory context), synthesize cross-cutting implications, and conclude with concrete recommendations. By reframing AI integration as a holistic sociotechnical problem rather than a purely technological one, we seek to bridge the gap between innovation and implementation and support accountable, equitable adoption. A summary of the challenges, proposed solutions, and policy implications discussed in this article is outlined in Table 1. We include a glossary of technical terms in Multimedia Appendix 2.

**Table 1.** Summary of challenges, proposed solutions, and policy implications.

Challenge	Proposed solutions (authors’ position)	Policy or practice implications
Technological		
Lack of explainability	Mandate regulatory-grade validation and audits and embed XAI^a^ principles for transparency	Require FDA^b^ or EMA^c^-style approval processes, develop explainability standards for health care AI^d^, fund XAI research, and require transparency reports
Algorithmic bias	Diverse and representative training data, debiasing techniques, and continuous auditing	Fund diverse dataset creation, institutionalize fairness audits, and enforce transparency in dataset composition
Lack of generalizability	Require multi-institutional validation, use of foundational models, and continuous monitoring	Make external validation mandatory and incentivize data sharing across institutions
Integration issues	Adopt interoperability standards (FHIR^e^, OMOP^f^, HL7^g^, DICOM^h^), modular API^i^-based design, and NLP^j^ for unstructured data	Promote universal interoperability standards and incentivize legacy system upgrades
Lack of multimodal AI	Invest in multimodal ML^k^ and HPC^l^ or cloud infrastructure and integrate structured and unstructured	Allocate funding for R&D^m^ and infrastructure and set guidelines for multimodal validation
Ongoing evolution	Continuous monitoring for drift, clinician-adjustable thresholds, embedded confidence scores, and fail-safe mechanisms	Mandate postdeployment auditing and regulate update protocols for “locked” versus adaptive algorithms
Difficulty of validation	Standardized validation frameworks, prospective RCTs^n^ for AI, and data repositories	Develop AI-specific RCT guidelines and fund longitudinal data repositories for validation
Human		
Resistance to change	Comprehensive training, education, and communication; frame AI as collaborative tool; and user-centered design	Develop national AI curricula for health care, incentivize adoption through training subsidies, and develop certification standards for AI usability
Lack of stakeholders’ involvement	Multidisciplinary development teams, “shadow clinician” immersion, and iterative feedback loops	Require co-design processes in procurement and implementation
Poor problem identification	Evidence-based prioritization and align AI initiatives with clinical and operational goals	Establish hospital AI governance committees with diverse stakeholder representation
Imbalanced human-AI interaction	Position AI as decision support, establish clear usage protocols, embed fail-safes, and training on limitations	Develop national guidelines on human oversight, set standards for AI confidence thresholds, and integrate AI interaction into clinical training
Education and training issues	Update medical curricula; tailored, role-specific training; and lifelong learning	Accredit AI in health courses and provide subsidies for continuing AI education
Lack of resources	Strategic funding and partnerships, train existing staff, phased implementation, and plan infrastructure before deployment	Allocate dedicated budgets for AI workforce and infrastructure, ensure equity in resource allocation, and incentivize public-private partnerships
Ethical or legal		
Liability concerns	Define legal responsibility across developers, providers, and institutions and regulatory approval for developers	Develop national and international liability frameworks for AI and clarify insurer responsibilities for AI-related claims
Data privacy and security	Strengthen consent models, data anonymization, and blockchain and advanced security	Strengthen HIPAA^o^ or GDPR^p^-style protections and enforce auditable consent processes
Informed consent issues	Mandate disclosure of AI use, dynamic consent models, and simplify explanations	Legally require patient notification of AI involvement and standardize consent forms for AI
Inequality in access	Ensure equity-driven design and subsidized deployment in underresourced settings; develop cost-effective, open-source AI; infrastructure investment; and diverse training data	Fund equitable AI deployment, mandate accessibility assessments for approved AI tools, policy frameworks to prioritize underserved populations
Conflict of interest	Mandatory transparency and disclosure, independent oversight bodies, and ethical training	Enforce strict conflict of interest declarations for procurement and research and require third-party evaluation
Regulation and compliance	Adaptive regulatory models and international regulatory harmonization	Create agile regulatory bodies and promote WHO^q^, FDA, or EMA coordination

a XAI: explainable artificial intelligence.

b FDA: Food and Drug Administration.

c EMA: European Medicines Agency.

d AI: artificial intelligence.

e FHIR: Fast Healthcare Interoperability Resources.

f OMOP: Observational Medical Outcomes Partnership.

g HL7: Health Level 7.

h DICOM: Digital Imaging and Communications in Medicine.

i API: application programming interface.

j NLP: natural language processing.

k ML: machine learning.

l HPC: high performance computing.

m R&D: research and design.

n RCT: randomized controlled trial.

o HIPAA: Health Insurance Portability and Accountability Act.

p GDPR: General Data Protection Regulation.

q WHO: World Health Organization.

## Technological Challenges

### Lack of Explainability

The lack of explainability in AI systems poses a major barrier to their integration into clinical workflows. Deep learning (a type of machine learning that uses artificial neural networks to learn from data, similar to the way we learn) models often operate as “black boxes,” producing predictions without transparent reasoning. This opacity limits clinicians’ ability to validate system outputs, undermining trust and accountability. When clinicians cannot trace the basis for a decision, potential errors or biases remain hidden, impeding clinical validation and adoption.

Lack of explainability is often misunderstood. Explainability does not require every model to be interpretable at the bedside; rather, it demands a traceable and auditable development process that documents how the model produces outcomes and how its reliability is verified. Given identical inputs, the system should produce reproducible outputs within a known error margin, and this process must be validated through rigorous testing.

Regulatory bodies such as the Food and Drug Administration (FDA) should develop validation and approval processes for AI systems analogous to those for drugs and medical devices, ensuring reliability and safety. At the same time, explainable AI techniques play a complementary role by offering interpretable insights—highlighting patient features that most influenced a prediction or generating visual explanations to support clinical review. The optimal pathway, therefore, combines rigorous validation to guarantee reproducibility with targeted transparency to build user trust. This balanced black box functionality at the point of care coupled with full traceability behind the scenes is essential for sustainable clinical integration.

### Algorithmic Bias

AI systems can produce biased outcomes that reflect and reinforce inequities in the underlying data. The sources of algorithmic bias are multifaceted and include historical inequities present in training datasets, socioeconomic disparities, and unequal access to health care services. Using unrepresentative data can lead to skewed model outputs that disadvantage certain populations. In health care, such bias may cause unequal care delivery and poorer outcomes for underrepresented groups. For example, a risk prediction tool used in US hospitals systematically underreferred Black patients to specialized care programs compared with White patients with equivalent medical needs. The algorithm used health care costs as a proxy for illness severity, assuming lower spending meant better health. In reality, lower expenditures reflected systemic barriers to care rather than improved outcomes, resulting in underallocation of resources to minority patients and amplifying inequities.

Reducing algorithmic bias requires more than technical adjustments. Models should be trained on datasets that are diverse and representative of the populations they serve. When such data are unavailable, methods such as resampling or data augmentation can be used to improve representativeness. However, these approaches must be applied cautiously, as they may introduce new biases if synthetic data reproduce flawed assumptions. Developers can also apply importance weighting to assign higher training value to underrepresented samples, improving model fairness.

Another critical step is eliminating proxy variables that encode protected attributes, such as race or socioeconomic status. Techniques such as blinding or data transformation can help obscure these attributes. Bias mitigation must also extend beyond the development phase: continuous auditing and multidisciplinary oversight involving ethicists, clinicians, and data scientists are necessary to identify emergent biases and sustain fairness.

A frequently cited parallel exists in non-AI health care systems, such as the revision of the estimated glomerular filtration rate formula, which until 2021 included race-based adjustments that were later removed to promote equity. AI systems, unlike static formulas, can adapt much faster once bias is detected, but only if the feedback mechanisms are properly implemented [5].

### Lack of Generalization

AI models often perform well in controlled research environments but struggle to replicate that performance in real-world clinical settings. This discrepancy arises because development and deployment environments differ in data quality, patient populations, and workflow practices. Real-world data are frequently noisy, incomplete, and inconsistent, leading to reduced reliability when AI systems are transferred between institutions. A model that functions effectively in 1 hospital may fail elsewhere due to variation in clinical protocols and documentation standards. The problem is compounded by the dynamic nature of health care, where evolving patient demographics and treatment practices can quickly outdate static models.

A notable example is IBM Watson for Oncology, one of the most widely publicized failures of AI in health care [6]. Trained primarily on synthetic data from a single cancer center, the system failed to generalize to diverse patient populations and local treatment practices. Its recommendations were inconsistent with clinical standards, leading to its withdrawal from the market. The Watson case underscores that training on narrow or synthetic datasets undermines generalizability and erodes trust in AI systems. To improve generalizability, models should be trained and validated on datasets from multiple institutions and populations, using both cross-validation and external validation techniques. Broader data sharing across health care systems remains essential, although challenging in practice. Continuous monitoring of deployed AI systems should become standard practice to detect performance drift early. Data quality, encompassing completeness, consistency, timeliness, and validity, is as critical as dataset size and should be prioritized equally. Clinician feedback during validation is indispensable, as practitioners provide contextual insights absent from retrospective data. Developing adaptive models capable of learning from new data and evolving clinical environments can further enhance reliability. Techniques such as site-specific fine-tuning, local threshold calibration, and transfer learning enable customization without sacrificing overall model robustness [7].

### Integration Issues

Health care environments often depend on diverse legacy systems and electronic health record (EHR) platforms, which rarely communicate seamlessly because each system uses distinct data formats, protocols, and standards. For AI systems to operate effectively, they must interact with these heterogeneous infrastructures. Achieving such interoperability is complex and resource-intensive owing to inconsistent protocols and data formats across platforms. The problem is further complicated by the nature of health care data itself, which includes structured fields, free-text clinical notes, imaging, and sensor data. Integrating these heterogeneous sources into unified, machine-readable formats remains a major technical and operational challenge.

Another barrier is ensuring real-time data exchange. Clinical workflows rely on up-to-date information, but legacy systems often cannot sustain the speed or reliability required for AI-assisted decision-making [8]. Compatibility gaps between AI applications and existing clinical software may require extensive customization and ongoing maintenance, making implementation resource-intensive and sometimes impractical.

Addressing these integration barriers requires coordinated technical and organizational strategies. Developers should assess existing technological infrastructure before deployment to ensure compatibility and minimize operational disruption. Standardized data models and exchange frameworks, such as the Observational Medical Outcomes Partnership and openEHR, provide structured approaches for harmonizing EHR data [9]. These frameworks enhance interoperability by creating common data structures that facilitate consistent data sharing and algorithm training across sites. Likewise, adopting interoperability standards such as Fast Healthcare Interoperability Resources enables structured data exchange across clinical systems, promoting real-time integration of AI tools into workflows. This not only improves compatibility and communication across platforms but also enhances the timeliness and reliability of clinical decision support.

### Lack of Multimodal AI

Most AI systems in health care still rely on a single data type, such as radiological images for disease detection. While unimodal systems have produced valuable insights, they often fail to capture the multidimensional nature of patient health. Modern health care generates diverse data sources, including clinical records, imaging, genomic sequences, wearable sensor data, and patient-reported outcomes, which together provide a more complete understanding of health and disease [10]. When AI models analyze only 1 modality, they risk overlooking critical context, such as comorbidities, lifestyle factors, or environmental influences, leading to incomplete or less accurate predictions. For example, chest computed tomography–based algorithms often struggle to differentiate overlapping pulmonary pathologies that could be better distinguished by integrating imaging with laboratory and clinical data.

Developing multimodal AI systems presents technical and operational challenges. Integrating heterogeneous data requires advanced architectures capable of aligning different data structures and ensuring consistent scaling and normalization. Combining structured and unstructured data also demands significant computational resources and data harmonization. These requirements highlight the need for investments in infrastructure and algorithmic innovation.

Improving multimodal AI capacity depends on sustained research investment in machine learning methods that can effectively process heterogeneous datasets. Standardization of data formats and the establishment of interoperable frameworks are essential to link imaging, text, and sensor-derived data. Expanding high-performance computing infrastructure, including cloud-based and distributed systems, will also be necessary to handle the scale of multimodal data. Ultimately, the integration of structured and unstructured clinical data with social, behavioral, and wearable inputs offers a path toward a holistic view of patient care. This approach promises improved diagnostic precision and personalized interventions, representing a key step in realizing the full potential of AI in health care.

### Ongoing Algorithmic Evolution

AI algorithms are designed to optimize their performance by continually evolving and learning new rules and techniques. While this adaptability offers potential advantages, it introduces serious risks when applied to health care. One concern is that evolving AI models may optimize for short-term metrics that do not align with patient well-being or clinical goals. For example, a predictive model might learn superficial correlations, such as associating positive outcomes with specific hospital equipment rather than genuine clinical indicators. Such behavior illustrates the danger of algorithms identifying statistical patterns that lack causal or clinical relevance.

The autonomous nature of adaptive AI also raises safety concerns. As these models explore new strategies, they may inadvertently adopt unvalidated or unsafe practices without adequate oversight. Another challenge involves data drift, the gradual change in input data distributions over time, which can degrade model performance even if the system was initially trained on large, heterogeneous datasets [11,12]. Changes in imaging protocols, scanner technology, or patient demographics can all contribute to declining accuracy, highlighting the need for continuous vigilance.

Mitigating these risks requires ongoing model monitoring rather than 1-time validation. Continuous surveillance should detect deviations from expected behavior and trigger corrective actions promptly. Structured feedback loops between clinicians and developers are essential to ensure that observed workflow disruptions or unexpected model behaviors inform retraining and improvement. Confidence assessment mechanisms should also be embedded directly into AI systems, preventing models from generating outputs when uncertainty exceeds safe thresholds. Fail-safe measures must be implemented to prevent unreliable predictions from influencing clinical decisions. Real-time monitoring for data drift is equally important; drift detection should enable dynamic recalibration of model thresholds to maintain performance under changing conditions.

### Difficulty of Validation of AI Efficacy

Validating the efficacy of AI systems in health care remains a major challenge. Robust validation demands real-world testing within clinical workflows while ensuring minimal disruption, a process that requires time, resources, and collaboration from health care professionals who may remain skeptical of emerging technologies. Establishing rigorous evidence through randomized controlled trials, long considered the gold standard for medical evaluation, is especially difficult for AI. Unlike static interventions, AI models evolve through continuous learning, complicating the definition of stable evaluation criteria. Additionally, ensuring data diversity and representativeness poses logistical and ethical barriers, as collaboration across multiple institutions and jurisdictions is often constrained by privacy regulations and data governance requirements. Retrospective datasets, which remain the predominant source for AI development, can introduce hidden biases, limiting the generalizability of findings to real-world clinical contexts.

To advance trustworthy AI, validation must be systematic and standardized. Data-sharing frameworks and cross-institutional collaborations should underpin the creation of centralized, anonymized repositories for longitudinal and prospective validation. Establishing regulatory and technical guidance for conducting AI-specific RCTs is crucial for ensuring that models undergo testing under real-world conditions. Co-developed validation protocols, jointly designed by developers, clinicians, and regulators, can help ensure clinical relevance and reliability. Transparency and reproducibility must also be enforced through open reporting of algorithms, datasets, and validation outcomes, enabling peer scrutiny and evidence-based refinement. Ultimately, regulatory endorsement by independent bodies such as the FDA and the World Health Organization should represent the benchmark for AI systems before clinical deployment, ensuring global credibility and patient safety.

## Human Factors

### Resistance to Change

Health care professionals’ resistance to AI can significantly impede its integration into clinical workflows. This resistance stems from several interrelated causes. Clinicians may hesitate to trust AI systems because of skepticism about their accuracy, reliability, and capacity to make sound clinical judgments. Such skepticism is often linked to a limited understanding of how AI systems function and concerns regarding transparency and explainability. Fear of accountability for adverse outcomes when relying on AI recommendations also contributes to reluctance. In addition, some professionals worry that AI may reduce their autonomy, challenge their expertise, or even replace their roles. The introduction of AI tools often disrupts established routines and requires adaptation to new processes, which can be perceived as burdensome and time-consuming. Moreover, clinicians express concern that AI could depersonalize care, diminishing empathy and the human connection central to medicine. Finally, institutional incentives and recognition programs can encourage adoption by aligning AI use with professional development and organizational goals (see sections Lack of Explainability, Education and Training Issues, Liability Concerns, Data Privacy and Security, Imbalanced Human-AI Interaction, Integration Issues).

### Lack of Stakeholders’ Involvement

The successful development and deployment of AI systems in health care depend on the early and sustained participation of diverse stakeholders, including clinicians, patients, administrators, data scientists, and IT professionals. When these groups are insufficiently engaged, integration efforts often encounter resistance, inefficiencies, and misaligned goals. Clinicians who are not included in development may distrust AI systems or find them poorly adapted to real-world workflows, resulting in limited adoption. Patient involvement is equally important, as transparency regarding data privacy, security, and ethical use helps build confidence and preserve autonomy. Administrators and technical teams ensure compliance with legal and infrastructure requirements, whereas their absence may lead to implementation failures or regulatory nonconformity. A lack of coordinated engagement can also create disconnects between developers who prioritize technical optimization and health care professionals who value usability and clinical outcomes.

Addressing these challenges requires formalized, multidisciplinary collaboration throughout design, testing, and deployment. Development teams should include health care providers, patients, ethicists, data scientists, and engineers to foster trust, ownership, and shared accountability. Immersive approaches, such as assigning “shadow clinicians,” in which developers or observers follow clinicians during daily practice, can offer valuable insights into workflow realities and user behaviors. These experiences inform the creation of intuitive interfaces that align closely with clinical routines and reduce cognitive load. Continuous feedback loops between clinicians, administrators, and AI developers should also be institutionalized to sustain improvement. Collaboration models such as the Google Health-Mayo Clinic partnership demonstrate that co-design and iterative engagement can significantly enhance usability, accuracy, and clinician acceptance [13] (see section Lack of Explainability).

### Poor Problem Identification and Prioritization

Health care organizations often face challenges in identifying and prioritizing where AI can deliver the most value. The sector’s complexity, with diverse patient needs and rapidly changing priorities, makes selecting suitable problems difficult. Successful integration requires alignment among clinicians, administrators, and IT professionals, each of whom may define value differently. Clinicians tend to emphasize diagnostic accuracy and clinical outcomes, administrators focus on cost efficiency and resource optimization, and IT teams prioritize feasibility and infrastructure compatibility. Misalignment among these perspectives can delay decision-making and impede adoption.

Resource limitations add further complexity. Many organizations face budget constraints, workforce shortages, and limited digital maturity, which hinder deployment. Prioritization must therefore balance clinical needs with technological readiness and available capacity. Poorly chosen initiatives risk diverting resources from higher impact opportunities, whereas flexible governance structures are essential to adapt priorities as clinical and operational contexts evolve. The COVID-19 pandemic exemplified this need for adaptability, as priorities shifted rapidly from long-term analytics to real-time applications such as outbreak tracking and hospital resource allocation [14]. The experience of IBM Watson for Drug Discovery illustrates how poor problem definition can undermine even advanced technologies. The project targeted a highly complex domain, drug discovery, where success rates are inherently low. Its broad objective produced results that were either redundant or not actionable, leading to limited adoption and eventual discontinuation [15].

Effective prioritization begins with multidisciplinary teams that combine clinical, technical, and administrative expertise. Evidence-based frameworks should guide the selection of use cases that address measurable clinical or operational needs. Projects that integrate seamlessly into workflows and demonstrate early, tangible impact are most likely to succeed and maintain stakeholder support. Continuous evaluation and real-world feedback enable organizations to refine focus areas and sustain alignment between AI initiatives and evolving health care demands.

### Imbalanced Human-AI Interaction

Determining the appropriate level of reliance on AI recommendations remains a key challenge in clinical practice. Overreliance can cause clinicians to accept outputs uncritically, neglecting clinical cues and diminishing independent judgment. This complacency risks diagnostic errors and weakens clinical reasoning skills over time. A well-documented example of this challenge is the Epic Sepsis Model developed by Epic Systems to identify patients at risk of sepsis. Many US hospitals deployed it, but several studies revealed that the tool frequently generated false alarms and missed a substantial number of true sepsis cases [16,17]. Clinicians who trusted it blindly risked patient harm, whereas those who ignored its alerts overlooked potential benefits. The episode underscored the importance of calibrated trust and clear human oversight in clinical AI use. Health care institutions must foster balanced human-AI interaction through 3 key strategies. First, AI systems should function strictly as decision support tools rather than autonomous decision-makers, ensuring that ultimate responsibility remains with clinicians. Second, comprehensive training programs should emphasize both the capabilities and limitations of AI, helping clinicians critically interpret system outputs and maintain accountability. Third, protocols and governance frameworks must delineate when and how to use AI, reserving complex or ethically sensitive decisions for human judgment. Finally, explainability remains vital: transparent models with interpretable outputs, such as visual saliency maps or confidence indicators, build user trust and encourage appropriate reliance. These measures ensure that AI augments rather than replaces clinical expertise, sustaining both safety and human-centered care (see sections Education and Training Issues, Regulation and Compliance Hurdles, and Lack of Explainability).

### Education and Training Issues

The successful adoption of AI technologies in health care depends heavily on clinicians’ ability to understand, interpret, and apply these tools effectively. However, many professionals lack sufficient training to do so. The absence of structured AI curricula in most medical education programs has created a knowledge gap that limits understanding of AI methodologies, capabilities, and limitations. This gap fosters hesitation and mistrust toward AI, as clinicians often feel uncertain about technologies they do not fully comprehend. Continuous professional development is also limited, as the rapid pace of AI innovation makes it difficult for institutions to maintain up-to-date training programs. Moreover, education rarely addresses the differing needs of health care roles. Physicians, nurses, administrators, and IT staff engage with AI in distinct ways, yet most training remains generic and insufficiently tailored to these contexts.

Effective solutions begin with reforming medical and health sciences education to include core AI competencies. Undergraduate and postgraduate curricula should incorporate foundational modules on AI methodology, data ethics, and computational reasoning, emphasizing practical applications and critical evaluation of AI outputs. Health care institutions should complement academic reform with ongoing, role-specific professional training that balances theoretical understanding with hands-on experience. Successful examples include dedicated AI workshops at Harvard Medical School, the Cambridge Center for AI in Medicine, and Weill Cornell Medicine–Qatar, which integrate interdisciplinary instruction and practical simulation. These initiatives demonstrate the value of combining technical and ethical education through cross-faculty collaboration. Cultivating a culture of continuous learning is equally essential: clinicians must be equipped to adapt to evolving technologies, reflecting the broader shift toward lifelong digital competence across the health workforce.

### Lack of Resources

Resource limitations, manpower, time, and finances pose significant barriers to integrating AI into clinical practice. Implementing AI systems often requires additional staff to manage data entry, monitoring, and tool maintenance. Most health care institutions already face staff shortages and heavy workloads, leaving little capacity for new responsibilities. The expert input required to train and validate AI algorithms is both expensive and time-consuming, as data labeling and annotation typically rely on highly skilled clinicians. These tasks divert specialists from clinical duties and increase operational costs. Maintaining AI systems further compounds the burden because updates, monitoring, and recalibration require sustained financial investment and technical expertise.

A well-documented example is the Google Health AI for diabetic retinopathy detection, which achieved strong research results but underperformed in real-world use due to resource constraints. In rural Thai clinics, limited access to high-quality imaging equipment and reliable internet connectivity reduced accuracy and slowed analysis. These limitations highlight that even well-designed systems depend on adequate infrastructure, training, and funding for successful deployment.

Overcoming resource constraints requires long-term strategic planning. Institutions should adopt phased implementation to distribute costs and workload, whereas policymakers establish funding mechanisms that support infrastructure, data curation, and workforce development. Public-private partnerships can accelerate progress by pooling resources and expertise. Finally, equitable resource allocation is essential to ensure that underresourced health care settings benefit from AI innovations, preventing the deepening of global health disparities.

## Ethical and Legal Considerations

### Liability Concerns

The integration of AI into health care has complicated traditional concepts of medical liability. When patient harm occurs following an AI recommendation, responsibility may be unclear—should it rest with the physician, the hospital, the developer, or the regulator who approved the system? Most jurisdictions still apply conventional tort law, assigning liability primarily to clinicians, even when decisions rely on algorithms that they neither designed nor fully understand. This creates uncertainty and discourages adoption.

Legal scholars have argued that hospitals should share responsibility under vicarious liability because physicians often operate under institutional authority. This approach simplifies adjudication and reflects the shared enterprise of AI-assisted care. However, current regimes rarely provide patients with a direct path to seek redress from developers (see section Regulation and Compliance Hurdles).

A pragmatic interim model is to assign joint liability to clinicians and hospitals, with insurers or institutional authorities covering claims. This balances accountability, promotes transparency, and provides legal certainty for health care professionals. Over time, national and international frameworks should evolve toward shared responsibility models that clearly delineate obligations across clinicians, institutions, and AI developers. Regulators should ensure that liability rules remain adaptive, reflecting the dynamic learning nature of AI systems and the distributed accountability inherent in their deployment.

### Data Privacy and Security

AI systems depend on vast amounts of sensitive patient data, raising major privacy and cybersecurity concerns. Large-scale data aggregation across EHRs, imaging repositories, and wearable devices increases exposure risks, making health care AI a prime target for breaches and unauthorized access. Research has shown that adversarial attacks can extract identifiable training data from AI systems, revealing patient information and compromising confidentiality. Even anonymized datasets are vulnerable, as sophisticated algorithms can reidentify individuals through pattern matching and cross-referencing. (Issues of data ownership and control further complicate the landscape, as patients, providers, and technology developers have competing interests in how medical data are used, shared, and monetized).

The 2016 DeepMind–Royal Free London NHS collaboration illustrates these challenges. Data from 1.6 million patients were shared without sufficient transparency or explicit consent, leading to significant public backlash when Google assumed direct control of the project [18]. This case underscored the risks of inadequate oversight in public-private partnerships and the urgent need for stronger governance.

Existing regulatory frameworks, such as Health Insurance Portability and Accountability Act in the United States and General Data Protection Regulation in Europe, remain insufficient to address AI-specific risks. Their compliance models were not designed for dynamic, data-intensive AI systems, and many countries have adopted them merely for market harmonization. We argue that next-generation frameworks must move beyond compliance checklists to embed proactive accountability and patient-centered control. This includes integrating medical confidentiality laws into AI data protection regimes, as seen in Qatar’s health legislation, which should serve as a model for linking data protection to ethical practice [19].

Emerging technologies such as blockchain can enhance traceability and ensure auditability of AI data use, improving both security and trust. However, without harmonized global data localization standards, cross-border AI operations will remain fragmented. A coordinated international approach, combining technical safeguards, legal harmonization, and ethical oversight, is essential for protecting privacy while enabling innovation in global health AI.

### Informed Consent Issues

Informed consent is a cornerstone of ethical medical practice, ensuring patients understand and agree to proposed interventions. However, integrating AI into health care complicates this principle. In several jurisdictions, clinicians are not legally required to inform patients when AI tools are used in diagnosis or treatment. This omission undermines transparency and can erode trust. The opacity of “black box” models makes it difficult for clinicians to explain how AI arrives at specific recommendations or decisions, further challenging patient comprehension. The dynamic nature of AI compounds the issue, as continuously learning systems evolve over time, potentially invalidating earlier explanations and raising questions about whether consent remains truly informed. There is also uncertainty about whether patients must reconsent when their data are reused to validate or refine AI systems (see sections Lack of Explainability and Ongoing Algorithmic Evolution).

To safeguard autonomy, informed consent laws should be amended to establish a universal baseline requiring that patients be notified whenever AI is used in their care. Patients must receive clear, comprehensible explanations about how AI systems operate, what data they use, and potential risks, including algorithmic bias and privacy concerns. Information should be provided through written materials, visual aids, or digital formats tailored to different literacy levels. Health care providers must be trained to communicate these concepts effectively, supported by developers who enhance explainability to facilitate such dialogue (see section Education and Training Issues).

Dynamic consent models offer a viable path forward by allowing patients to update preferences over time and choose specific permissions for data use, including training, validation, and development. Patients should also retain the right to refuse AI involvement in their care altogether. Regulators must enforce standardized consent guidelines to ensure patients are informed consistently, regardless of jurisdiction. In institutional settings where explicit consent is impractical, such as hospitals using AI-based tracking, implied consent may apply, but clear notification should remain mandatory. Establishing hospital ethics committees focused on AI oversight would help ensure ethical compliance and accountability.

### Inequality in Access to Health Care

AI has the potential to widen existing disparities in health care access. Underresourced clinics and hospitals, especially in rural or low-income areas, often lack the infrastructure required for AI adoption, including reliable internet connectivity, high-performance computing, and trained personnel. This digital divide limits their ability to implement AI systems, creating inequities in access to advanced diagnostics and personalized care. Moreover, many AI models are trained on datasets that underrepresent minority populations, leading to biased performance and diagnostic inaccuracies for these groups. Such biases can reinforce structural inequities, erode trust, and result in poorer outcomes for marginalized communities. The high cost of developing and maintaining AI systems further exacerbates disparities, allowing well-funded institutions to deploy advanced tools while resource-limited facilities fall behind.

Ensuring equitable access requires deliberate and globally coordinated action. AI developers must prioritize designing cost-effective, scalable, and open-source models that can operate on basic hardware and limited bandwidth. Training datasets must be diverse and representative to minimize bias and enhance performance across populations. Governments and nonprofit organizations should allocate targeted funding to strengthen digital infrastructure and workforce capacity in low-resource settings, whereas public-private partnerships can facilitate technology transfer and cost-sharing mechanisms. Policymakers should also adopt proactive regulatory measures that mandate equitable distribution of AI resources, ensuring that underserved regions are prioritized in national digital health strategies. Through these efforts, AI can evolve from a technology that risks deepening inequity into a tool that actively promotes fairness, inclusivity, and universal access to high-quality care.

### Conflicts of Interest

Conflicts of interest in AI arise when the personal, financial, or institutional interests of health care providers, developers, or policymakers compromise patient-centered decision-making. Financial relationships between clinicians and AI companies can bias procurement and use, as physicians involved in AI development may favor tools in which they hold investments or royalties. Partnerships between developers and health care institutions can also blur boundaries between clinical integrity and commercial gain, particularly when companies sponsor trials or offer incentives that influence results or adoption decisions. The collapse of Babylon Health, a UK startup once valued at $4.2 billion, illustrates the dangers of unchecked conflicts. Despite early concerns from regulators and researchers, Babylon secured NHS contracts and political endorsements through lobbying and financial contributions [20]. Its rapid expansion, driven more by political and financial support than by proven clinical efficacy, ended in bankruptcy in 2023, eroding public trust and wasting substantial investment. The case highlights how opaque financial and political relationships can distort AI adoption, undermining evidence-based medicine and public confidence.

Mitigating such risks requires strict transparency and governance measures. Mandatory disclosure of all financial ties between clinicians, institutions, and AI developers should be a regulatory requirement. Hospitals should establish internal ethics committees to oversee AI procurement and usage decisions, ensuring these are guided by patient safety and clinical benefit rather than commercial interest. Independent auditing of AI trials and deployment outcomes should be routine to identify potential biases. Finally, separating the roles of developers and clinical decision-makers, through independent evaluation bodies, would safeguard neutrality and promote integrity in AI adoption.

### Regulation and Compliance Hurdles

The absence of unified, AI-specific regulation remains one of the most significant barriers to safe and effective implementation of AI in health care. Existing laws, while robust in data protection, do not adequately address the dynamic and adaptive nature of AI, creating uncertainty around liability, validation, and accountability. Regulatory fragmentation compounds these challenges. For example, US frameworks such as HIPAA and the FDA approval processes differ markedly from the EU’s GDPR and the AI Act, resulting in conflicting expectations and inconsistent oversight for developers and health care providers [20]. In countries lacking formal approval mechanisms, startups may find themselves in regulatory limbo, unable to bring products to market or compete with foreign firms holding recognized certifications. At the same time, opaque “black box” models and the absence of standardized evaluation metrics make it difficult for regulators to ensure safety and reliability across diverse health care contexts.

We argue that an adaptive and collaborative regulatory framework is urgently required. This should prioritize (1) international harmonization, promoting coordination between bodies such as the World Health Organization, the FDA, the European Medicines Agency, and regional regulators to develop common AI validation and approval standards; (2) agile regulation, establishing rapid-update mechanisms that can evolve alongside technological innovation while preserving patient safety; and (3) transparent oversight, mandating algorithmic explainability, performance auditing, and public reporting of outcomes. Additionally, regulators must embed context-aware evaluation, ensuring that assessments reflect real-world clinical variation rather than controlled laboratory conditions. By embracing adaptive, risk-based regulation, policymakers can safeguard innovation while maintaining accountability and equity in global health care AI deployment.

## Conclusions

The integration of AI into health care holds transformative potential but remains hindered by complex technological, human, and ethical challenges. Despite promising advances, real-world adoption has been slow, with successful implementations still the exception rather than the norm. This paper provides a holistic framework categorizing the major challenges, technological, human, and ethical or legal, and presents evidence-informed, actionable strategies to address them.

From a technological perspective, progress depends on explainable, unbiased, and interoperable systems that can generalize across populations and settings. Human challenges, including resistance to change, insufficient stakeholder engagement, poor problem definition, and limited training, must be addressed through education, collaboration, and resource investment. Ethical and legal barriers, including liability, privacy, consent, inequality, conflicts of interest, and fragmented regulation, require adaptive governance and robust safeguards to protect patient rights while enabling innovation.

In practice, we prioritize actions in three tiers: first, governance and safety; second, workflow and people; and third, equity and scale. This translates to institutional oversight and validation parity with postmarket monitoring stakeholder co-design and role-specific education for fit-for-workflow and calibrated human-AI interaction, and equity through interoperability and infrastructure in low-resource settings, diverse datasets, and cross-jurisdictional coordination.

The path forward demands sustained, interdisciplinary collaboration among clinicians, developers, regulators, and policymakers. AI must be viewed not as a technological end point but as a continually evolving tool within a sociotechnical ecosystem. By combining transparent design, equitable access, and adaptive regulation, health care systems can move beyond hype to responsible implementation. Realizing AI’s full potential will depend not only on technical breakthroughs but also on collective commitment to ethics, accountability, and human-centered care.

## Supplementary material

10.2196/70921 Multimedia Appendix 1 Detailed technical explanations, case examples, and jurisdictional analyses.

10.2196/70921 Multimedia Appendix 2 Glossary of technical terms.

## Abbreviations

AI: artificial intelligence
EHR: electronic health record
