# Supplementary material for: Hype vs Reality in the Integration of Artificial Intelligence in Clinical Workflows
Source: JMIR Form Res. 2025 Dec 12;9:e70921. doi: 10.2196/70921 (PMC12700513; doi:10.2196/70921)
Supplement: Multimedia Appendix 1 [file formative-v9-e70921-s001.docx]

This file should be uploaded as **Supplementary File 1** accompanying the Viewpoint manuscript titled ‘Hype vs Reality: Integration of Artificial Intelligence in Clinical Workflows.'

# Appendix A. Expanded Supplementary Material

## Overview

To maintain concision consistent with the Viewpoint format, this appendix expands upon the examples, contextual details, and methodological considerations that informed the core analysis in the main manuscript. It integrates technical, human, and ethical perspectives that were abridged during manuscript condensation. The appendix is structured across three domains—Technological, Human Factors, and Ethical & Legal—each containing detailed illustrative material and synthesis.

## Table A1. Summary of Supplementary Material

| Domain | Subsection | Supplementary Content Summary |
| --- | --- | --- |
| Technological | Lack of Explainability | Detailed overview of interpretability frameworks and clinical validation analogies; illustrative examples of explainable AI (XAI). |
| Technological | Algorithmic Bias | Expanded discussion of bias mitigation methods and the eGFR recalibration case. |
| Technological | Lack of Generalization | IBM Watson Oncology case study and overview of adaptive learning frameworks. |
| Human Factors | Resistance to Change | Survey data on clinician attitudes and historical adoption experiences. |
| Human Factors | Stakeholder Involvement | Participatory design and 'shadow clinician' fieldwork examples. |
| Human Factors | Problem Prioritization | Resource-allocation and governance examples during COVID-19. |
| Ethical & Legal | Liability & Accountability | Jurisdictional comparison of AI liability frameworks and shared-responsibility proposals. |
| Ethical & Legal | Data Privacy and Security | HIPAA–GDPR comparison and lessons from data-breach cases. |
| Ethical & Legal | Informed Consent | Dynamic-consent platform case studies in the UK and Qatar. |
| Ethical & Legal | Inequality & Access | Global digital divide metrics and strategies for equitable AI access. |
| Cross-Cutting | Regulation & Compliance | Comparative analysis of FDA, EMA, and PMDA regulatory pathways. |

## A1. Technological Domain

### A1.1 Lack of Explainability

Explainability remains a defining challenge in clinical AI implementation. While traditional algorithms can be audited through explicit equations or logic trees, modern deep learning models—particularly convolutional and transformer-based architectures—are largely opaque to end-users. This opacity undermines trust, limits accountability, and complicates regulatory review. Two major schools of thought frame explainability: intrinsic interpretability (where models are inherently transparent) and post-hoc explainability (where explanations are derived after prediction). Examples of the latter include SHAP, LIME, and saliency mapping for medical imaging. Clinically, these methods serve an analogous role to laboratory validation—each interpretation must demonstrate reliability before informing patient care. Yet, no standardized metrics exist for “sufficient” explainability across contexts. Future research should emphasize human-centered interpretability benchmarks co-designed with clinicians.

### A1.2 Algorithmic Bias

Bias arises when training data fail to represent the population served, leading to unequal model performance across demographic or socioeconomic groups. Mitigation begins with improved data sampling strategies, synthetic augmentation, and fairness-aware training objectives. The eGFR recalibration case exemplifies how embedded racial adjustments can perpetuate inequity. Originally developed using race-specific modifiers, eGFR models systematically overestimated kidney function in Black patients, delaying referrals and treatment. The 2021 consensus to eliminate race as a variable represents a landmark shift toward equity-driven recalibration in clinical AI.

### A1.3 Lack of Generalization

The failure of models to generalize beyond their training environment remains a major impediment to real-world adoption. AI systems trained on homogeneous datasets often falter when applied in different hospitals or demographic settings. The IBM Watson for Oncology project is emblematic: despite early publicity, it struggled to replicate results outside trial hospitals due to limited contextual adaptation and reliance on curated expert rules. Generalization depends on diversified datasets and continuous validation pipelines. Adaptive learning frameworks—where models update incrementally while preserving baseline performance—show promise but require regulatory mechanisms to monitor post-deployment drift.

## A2. Human Factors Domain

### A2.1 Resistance to Change

Clinician resistance to AI stems from perceived threats to autonomy, skepticism about accuracy, and uncertainty over liability. Surveys show lower adoption where AI systems lack transparency or demonstrable clinical benefit. Early AI triage tools in emergency departments faced rejection when clinicians felt excluded from development stages. Overcoming resistance requires aligning technology with professional identity. When framed as decision support rather than replacement, AI is more readily accepted. Implementation strategies should include peer-led training and formal recognition of AI proficiency.

### A2.2 Stakeholder Involvement

Multidisciplinary engagement throughout AI design enhances trust and usability. Participatory design frameworks integrate clinicians, patients, ethicists, and engineers. The Stanford Radiology shadow clinician model exemplifies this approach: developers shadowed radiologists during normal workflow to identify friction points and co-design interfaces aligned with cognitive flow. Such involvement fosters ownership and ensures algorithms augment rather than disrupt practice.

### A2.3 Problem Prioritization

Misaligned problem selection undermines AI’s impact. Healthcare leaders often prioritize high-visibility projects over high-need areas. The COVID-19 pandemic underscored agile prioritization: AI resources were redirected toward outbreak modeling, ventilator allocation, and diagnostic triage. Effective prioritization depends on clear clinical metrics, stakeholder consensus, and balance between feasibility and readiness.

## A3. Ethical and Legal Domain

### A3.1 Liability and Accountability

The diffusion of responsibility in AI-assisted care complicates traditional medical liability. Physicians remain primary bearers of responsibility even when influenced by algorithms. Legal scholars propose shared liability among clinicians, hospitals, and developers. Emerging frameworks in the EU, Singapore, and Japan advocate adaptive liability linking responsibility to AI development stages. Transparent audit trails documenting inputs, outputs, and version histories can facilitate accountability.

### A3.2 Data Privacy and Security

Healthcare AI relies on large datasets, amplifying exposure to breaches. HIPAA and GDPR emphasize consent and minimization but fail to address dynamic learning models. The DeepMind–Royal Free NHS case demonstrates how insufficient transparency erodes trust. Blockchain and federated learning offer decentralized approaches to maintain auditability without compromising privacy. A harmonized global framework linking privacy regulation with algorithmic accountability is essential.

### A3.3 Informed Consent

AI complicates informed consent as patients may not know algorithms contribute to care. Dynamic consent allows updating preferences and specifying data reuse. Implementations in the UK NHS and Qatar show that transparent communication increases trust. Ethical AI deployment requires integrating consent models into governance frameworks to ensure continuous transparency.

### A3.4 Inequality and Access

Unequal digital infrastructure creates a two-tier AI landscape. Low-resource settings lack connectivity, hardware, and expertise, limiting access. Datasets often exclude underrepresented groups, perpetuating bias. Solutions must include open-source, resource-efficient models and targeted funding to reduce disparities.

### A3.5 Regulation and Compliance

AI-specific regulation is fragmented globally. While the FDA, EMA, and PMDA have guidance on adaptive algorithms, static premarket reviews remain the norm. A harmonized, risk-based regulatory approach with post-market surveillance, monitoring, and transparency is needed. International harmonization led by WHO or OECD would accelerate safe adoption while maintaining accountability.

## References

A complete JMIR-style reference list (1–30) accompanies this appendix in the final document, containing all cited materials consistent with the main manuscript.

# References

Note: This supplementary appendix summarizes extended examples and contextual discussion; references are provided in full below without in-text numbering for readability.

1. Amann J, Blasimme A, Vayena E. Explainability for artificial intelligence in healthcare: a multidisciplinary perspective. BMC Med Inform Decis Mak. 2020;20(1):310.

2. Tonekaboni S, Joshi S, McCradden MD, Goldenberg A. What clinicians want: contextualizing explainable machine learning for clinical end use. NPJ Digit Med. 2019;2:102.

3. Mehrabi N, Morstatter F, Saxena N, et al. A survey on bias and fairness in machine learning. ACM Comput Surv. 2021;54(6):1–35.

4. Diao JA, et al. Clinical implications of removing race from estimates of kidney function. JAMA. 2021;325(2):184–186.

5. Strickland E. IBM Watson, heal thyself. IEEE Spectrum. 2019;56(4):24–31.

6. Rajpurkar P, Chen E, Banerjee O, Topol E. AI in health and medicine. Nat Med. 2022;28(1):31–38.

7. Longoni C, Bonezzi A, Morewedge CK. Resistance to medical artificial intelligence. J Consum Res. 2019;46(4):629–650.

8. O’Sullivan D, et al. Clinician perspectives on AI in healthcare: barriers and facilitators. BMJ Health Care Inform. 2022;29(1):e100622.

9. Cai CJ, et al. Human-centered tools for coping with imperfect algorithms during clinical decision-making. CHI Conf Proc. 2019:1–14.

10. Blease C, et al. Artificial intelligence and the future of psychiatry. Psychol Med. 2019;49(9):1429–1436.

11. Cabitza F, et al. Unintended consequences of machine learning in medicine. JAMA. 2017;318(6):517–518.

12. Liang H, Tsui BY, Ni H, et al. Evaluation and accurate diagnoses of pediatric diseases using AI. Nat Med. 2019;25(3):433–438.

13. Mittelstadt BD. Principles alone cannot guarantee ethical AI. Nat Mach Intell. 2019;1(11):501–507.

14. Floridi L, Cowls J. A unified framework of five principles for AI in society. Harv Data Sci Rev. 2019;1(1):1–15.

15. Price WN II, Cohen IG. Privacy in the age of medical big data. Nat Med. 2019;25(1):37–43.

16. Royal Free NHS Foundation Trust. DeepMind health data agreement review. 2017.

17. Rieke N, et al. The future of digital health with federated learning. NPJ Digit Med. 2020;3(1):119.

18. Chassang G. The impact of the EU general data protection regulation on scientific research. Ecancermedicalscience. 2017;11:709.

19. Kaye J, et al. Dynamic consent: a patient interface for twenty-first century research networks. Eur J Hum Genet. 2015;23(2):141–146.

20. El Emam K, et al. Consent and anonymization in data sharing for health research. J Law Med Ethics. 2011;39(2):240–251.

21. Leslie D. Tackling COVID-19 through responsible AI innovation. Nat Mach Intell. 2020;2(8):404–406.

22. Obermeyer Z, et al. Dissecting racial bias in an algorithm used to manage population health. Science. 2019;366(6464):447–453.

23. Brall C, Berlin C, Kenett RS. Equitable AI for global health. Bull World Health Organ. 2022;100(9):574–584.

24. Jhala S, et al. AI-driven healthcare in low-resource settings. Front Digit Health. 2022;4:875649.

25. Goodman KW. Ethics, medicine, and information technology. Cambridge University Press; 2021.

26. Dignum V. Responsible artificial intelligence: how to develop and use AI in a responsible way. Springer; 2019.

27. Wang F, Kaushal R, Khullar D. Should health care demand interpretable artificial intelligence or accept “black box” medicine? Ann Intern Med. 2020;172(1):59–60.

28. Hatherley JJ. Limits of trust in medical AI. J Med Ethics. 2020;46(7):478–481.

29. Wiegand T, et al. WHO guidance on the ethics and governance of AI for health. Geneva: World Health Organization; 2021.

30. European Commission. Proposal for a Regulation on Artificial Intelligence. Brussels: European Union; 2021.
