# Supplementary material for: Hype vs Reality in the Integration of Artificial Intelligence in Clinical Workflows
Source: JMIR Form Res. 2025 Dec 12;9:e70921. doi: 10.2196/70921 (PMC12700513; doi:10.2196/70921)
Supplement: Multimedia Appendix 2 [file formative-v9-e70921-s002.docx]

Appendix 2

**Glossary of Technical Terms**

| **Term** | **Definition** | **Plain-language Explanation** |
| --- | --- | --- |
| **Importance weighting technique** | A statistical method that gives more influence to underrepresented cases in a dataset. | Helps AI pay more attention to rare or minority cases so it learns effectively. |
| **Resampling / Data augmentation** | Methods to artificially increase data diversity by duplicating, modifying, or generating new examples. | Like making extra copies or variations of patient data so AI sees more balance. |
| **Synthetic data** | Artificially generated data created by algorithms rather than collected from real patients. | Fake patient records made by computers to train AI when real data is scarce. |
| **Proxy variables** | Variables that indirectly reveal sensitive attributes like race, gender, or income. | Data that indirectly gives away sensitive info, e.g., postal code reveals income. |
| **Blinding** | A method of hiding certain variables from an AI model during training. | Covering up info that might cause bias so the AI can’t use it in decisions. |
| **Privacy-preserving ML / Federated learning** | Methods that allow AI to be trained across multiple institutions without moving raw data. | The AI model travels to hospitals, learns locally, then combines knowledge—keeping data private. |
| **Cross-validation / External validation** | Techniques for testing an AI model on new sets of data to check generalizability. | Like giving a student practice exams from different schools to check real learning. |
| **Adaptive AI / Continuous learning** | AI models that update themselves as new data comes in. | AI that keeps learning over time so it stays up to date with new data. |
| **Transfer learning** | Using a model trained on a large dataset and fine-tuning it for a smaller, specific dataset. | Like a doctor trained in general medicine who later specializes in cardiology. |
| **Foundational models** | Large AI models trained on vast datasets that can be adapted to many tasks. | A general-purpose AI model that can be customized for specific hospital needs. |
| **OMOP** | A standardized framework for structuring and sharing health records. | A common template for hospitals so electronic records ‘speak the same language’. |
| **openEHR** | An open standard for storing and exchanging electronic health records. | A universal format for medical records so systems can work together easily. |
| **FHIR** | A standard for exchanging healthcare information electronically. | A set of rules allowing hospital IT systems to share information smoothly. |
| **IHE** | An initiative to improve how healthcare IT systems exchange information. | Guidelines that help medical software and systems work together better. |
| **HL7 / DICOM** | Standards: HL7 for clinical text data, DICOM for medical imaging data. | HL7 makes text data shareable; DICOM makes imaging (X-rays, MRIs) shareable. |
| **Data harmonization** | The process of cleaning and standardizing data so it can be combined and analyzed. | Making sure hospitals record info the same way, e.g., ‘heart attack’ vs. ‘MI’. |
| **Natural Language Processing (NLP)** | AI that enables computers to understand and analyze human language. | Lets AI read and understand doctors’ notes, patient records, or research papers. |
| **High-performance computing (HPC), GPUs, TPUs** | Advanced hardware and cloud systems for processing very large datasets. | Supercomputers or chips that give AI the power to run complex models. |
| **Multimodal AI** | AI that combines multiple types of data (e.g., imaging, labs, notes) in one system. | Instead of only X-rays, AI also considers labs and history for a fuller picture. |
| **Algorithmic drift / Data drift** | When an AI model’s performance declines due to changes in the new data it sees. | Like a doctor who hasn’t updated knowledge—AI gets outdated if not monitored. |
| **Confidence thresholds** | The probability level at which an AI model decides to make a prediction. | A safety setting that controls how ‘sure’ AI must be before raising an alert. |
| **Explainable AI (XAI)** | Techniques that make AI decision-making more transparent and interpretable. | Tools that let clinicians see why AI made a decision, e.g., what part of an X-ray it used. |
